# Focused ultrasound in modern medicine: bioengineering interfaces, molecular effects, and clinical breakthroughs

**DOI:** 10.3389/fbioe.2025.1610846

**Published:** 2025-08-29

**Authors:** Xia Li, Yi Liu

**Affiliations:** Yangtze River Shipping General Hospital, Wuhan, Hubei, China

**Keywords:** ultrasound, molecular mechanisms, damage, high-intensity focused ultrasound (HIFU), thrombosis

## Abstract

Ultrasound technology, first utilized in 1947–1948 for diagnostic applications in obstetrics and gynecology, has significantly expanded its scope to include both diagnostic and therapeutic uses in modern medicine. The advent of continuous therapeutic ultrasound has allowed for its application in treating musculoskeletal pathologies, enhancing fracture healing, and even facilitating tumor treatment when paired with MRI. Ultrasonic cavitation, gas body activation, and mechanical stress are primary non-thermal mechanisms responsible for its biological effects. Recent advancements have expanded ultrasound’s potential to enhance drug delivery, as seen in the sonoporation phenomenon, where ultrasound triggers cell membrane permeability. This process can be reversible or irreversible, offering exciting possibilities for targeted treatments. Additionally, microbubbles are used to intensify US-induced effects, contributing to therapeutic applications such as high-intensity focused ultrasound (HIFU) for cancer ablation and drug delivery. Molecular ultrasound imaging, which incorporates microbubbles targeted to specific biomarkers, allows for the non-invasive visualization of molecular processes such as angiogenesis, inflammation, and thrombosis. This capability holds significant promise for early disease detection and monitoring, particularly in cancer and cardiovascular conditions. The aim of this review is to explore the diverse molecular mechanisms underlying ultrasound’s therapeutic and diagnostic capabilities, assess its potential for improving patient outcomes, and highlight the future directions for clinical integration of ultrasound in medicine.

## 1 Introduction

Ultrasound technology has rapidly evolved from a diagnostic imaging modality into a multifaceted therapeutic tool capable of precise tissue targeting and molecular modulation. In the contemporary medical landscape, ultrasound-based therapies—including high-intensity focused ultrasound (HIFU), extracorporeal shockwave lithotripsy, and ultrasound-mediated drug delivery—are increasingly recognized for their ability to induce biological effects that extend beyond traditional imaging (Gliklich et al., 2007; Ninet et al., 2005; Zini et al., 2012; Taran et al., 2009; Kaneko and Willmann, 2012; Deshpande et al., 2010). The therapeutic potential of ultrasound lies in its capacity to deliver focused energy deep within tissues, achieving site-specific intervention while minimizing collateral damage to surrounding structures. This selectivity is central to modern approaches that seek to leverage physical energy for minimally invasive, yet highly effective, clinical interventions.

**FIGURE 1:**
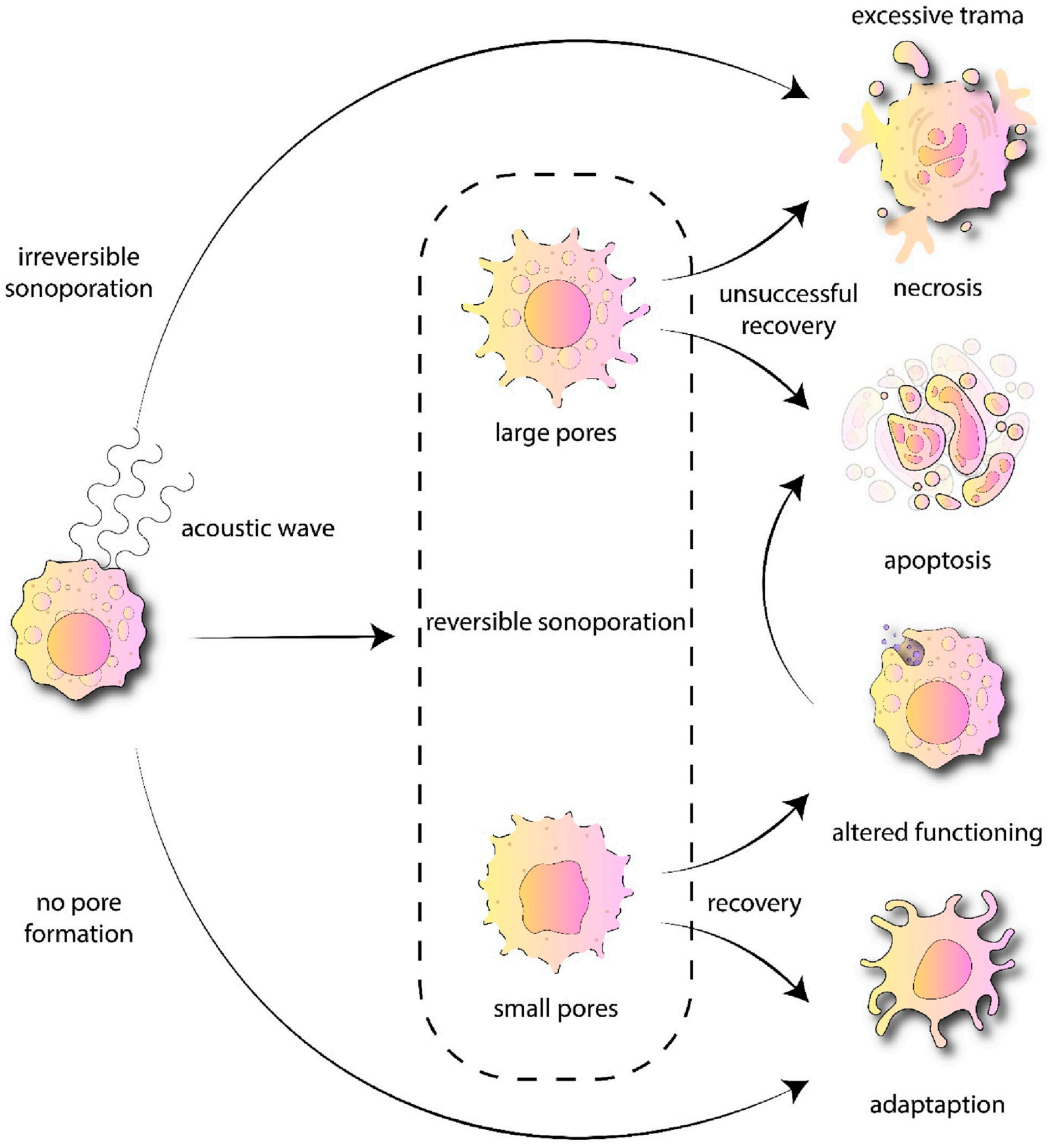
Showing possible cellular impacts resulting from sonoporation. As the sound wave approaches the cell, it causes the cell membrane to form openings of varying sizes. Necrosis happens when the membrane is unable to be repaired and the cell damage is too extensive to heal, leading to permanent sonoporation. Conversely, if the cell is able to recuperate following exposure to ultrasound (US), this might change its functioning, and if it cannot adapt, apoptosis will be triggered.

The underlying mechanisms by which ultrasound exerts therapeutic effects are diverse and depend on the mode, frequency, and intensity of the energy delivered (O'Brien, 2007). At the molecular level, ultrasound can enhance cell membrane permeability through processes such as sonoporation, enabling the targeted delivery of therapeutic agents into cells (Bess, 2023; Awal et al., 2021) (Figure 1). Additionally, the thermal and mechanical effects of focused ultrasound can induce protein denaturation, tissue ablation, and stimulation of tissue regeneration, making it a versatile platform for treating conditions ranging from solid tumors to musculoskeletal injuries (Kremkau, 1979). These molecular and cellular responses are the result of complex biophysical interactions, including acoustic cavitation, radiation force, and localized heating, all of which can be harnessed to modulate biological systems in a controlled manner.

Recent advancements in ultrasound technology have greatly expanded its range of clinical applications. For example, the combination of ultrasound with imaging modalities such as MRI has facilitated precise targeting for both benign and malignant tumors, improving treatment outcomes and reducing side effects (Gliklich et al., 2007; Ninet et al., 2005; Zini et al., 2012; Taran et al., 2009; Kaneko and Willmann, 2012; Deshpande et al., 2010). The integration of ultrasound with molecular techniques has further broadened its potential, allowing for the development of novel strategies in areas such as gene therapy and immunomodulation (Gheorghe et al., 2023; Sun S. et al., 2022). Moreover, focused ultrasound is being explored as a means of enhancing the efficacy of conventional therapies. By temporarily increasing vascular and cellular permeability, ultrasound can improve the distribution and uptake of chemotherapeutic drugs, as well as biological agents used in immunotherapy. This synergistic approach aims to maximize therapeutic benefit while reducing systemic toxicity—a longstanding challenge in cancer treatment and other chronic diseases (Miller et al., 2012; Bader et al., 2025; Laganà et al., 2024).

While the early use of ultrasound in medicine can be traced back to 1947–1948, when Karl Dussik and his brother Friederick introduced hyperphonography for visualizing cerebral ventricles (Shung, 2011), its therapeutic applications have advanced remarkably in recent decades. Initially, ultrasound gained widespread acceptance in diagnostic imaging due to its safety, affordability, and portability. It became a mainstay for monitoring pregnancies, guiding joint injections, and identifying soft tissue disorders (Analan et al., 2015; Robertson and Baker, 2001). However, as engineering innovations enabled more precise control of ultrasound energy, the technology found new roles in clinical interventions. The introduction of extracorporeal shockwave lithotripsy in the 1980s revolutionized the treatment of kidney stones, and HIFU has since emerged as a promising modality for non-invasive tumor ablation and the treatment of prostate cancer (Yu and Xu, 2014; Lentacker et al., 2014; Wang et al., 2017; Boissenot et al., 2016).

Despite these successes, therapeutic ultrasound is not without challenges. The biological effects of ultrasound are influenced by numerous factors, including tissue composition, energy dose, and treatment duration. Tissues with higher protein content, such as muscle, tend to absorb mechanical energy more efficiently than those with higher water content, such as fat, while bone primarily reflects ultrasound waves due to its density and impedance mismatch (O'Brien, 2007; Bess, 2023; Awal et al., 2021). Unintended bioeffects—including thermal injury and mechanical damage—underscore the importance of standardized protocols, dosimetry, and rigorous safety assessments in clinical practice (Miller et al., 2012; Bader et al., 2025).

While ultrasound-mediated delivery has attracted significant attention for its non-invasive and targeted capabilities, other bioengineering-based methods are also being explored for therapeutic applications. Electroporation, for example, uses brief electric pulses to transiently permeabilize cell membranes, thereby enhancing the delivery of drugs or genetic material (Jacobs et al., 2025; Balantič et al., 2021; Rubinsky, 2007). Although highly effective for gene transfer, electroporation is generally limited to localized or superficial applications and may cause significant discomfort or tissue damage in some settings (Young and Dean, 2015; Mahnič-Kalamiza and Miklavčič, 2022; Choi et al., 2022). Nanoparticle-mediated therapies offer another promising approach, enabling the encapsulation and targeted delivery of therapeutics with high specificity; however, challenges remain regarding biodistribution, long-term safety, and clearance (Li X. et al., 2024; Verma et al., 2023). Compared to these methods, ultrasound-based administration offers the advantage of deep tissue penetration, real-time imaging guidance, and the ability to combine with microbubbles or nanocarriers for spatiotemporally controlled release.

The objective of this review is to explore the molecular mechanisms underlying therapeutic ultrasound, assess its diverse clinical applications, and evaluate its impact on patient care. By bridging the gap between basic biophysical principles and practical clinical outcomes, we aim to highlight how ultrasound technology is shaping the future of precision medicine and transforming the therapeutic landscape. The central hypothesis guiding this work is that ultrasound, beyond its well-established diagnostic utility, can be harnessed to induce beneficial molecular changes—enabling the treatment of disease, enhancement of tissue repair, and targeted drug delivery. In doing so, we seek to provide a comprehensive overview that not only summarizes current knowledge but also identifies future directions for research and clinical practice.

## 2 A mechanistic overview about ultrasound

Ultrasound energy is a powerful method for producing biological impacts. With enough understanding of the causes and measurement of exposure, biological effects can be either utilized for treatment or prevented in diagnostic settings. In medical treatments, ultrasound can produce effects by generating heat or through nonthermal techniques such as inducing ultrasonic cavitation, triggering gas bubbles, applying mechanical stress, or utilizing other unexplained nonthermal processes (Nyborg et al., 2002). From a diagnostic viewpoint, ultrasound is generally produced by a piezoceramic emitting brief pulses, which typically contain 1–5 cycles. Diagnostic ultrasound is commonly characterized by the primary frequency of its sound waves, which typically falls within the 2–12 MHz range. The thickness of the ceramic in the device is often associated with its frequency. Devices for therapeutic ultrasound can utilize either intermittent pulses or steady waves to transmit efficient ultrasonic energy to tissues. Certain devices function at increased amplitude, which often results in the generation of shocked or distorted waves (Miller et al., 2012).

**FIGURE 2:**
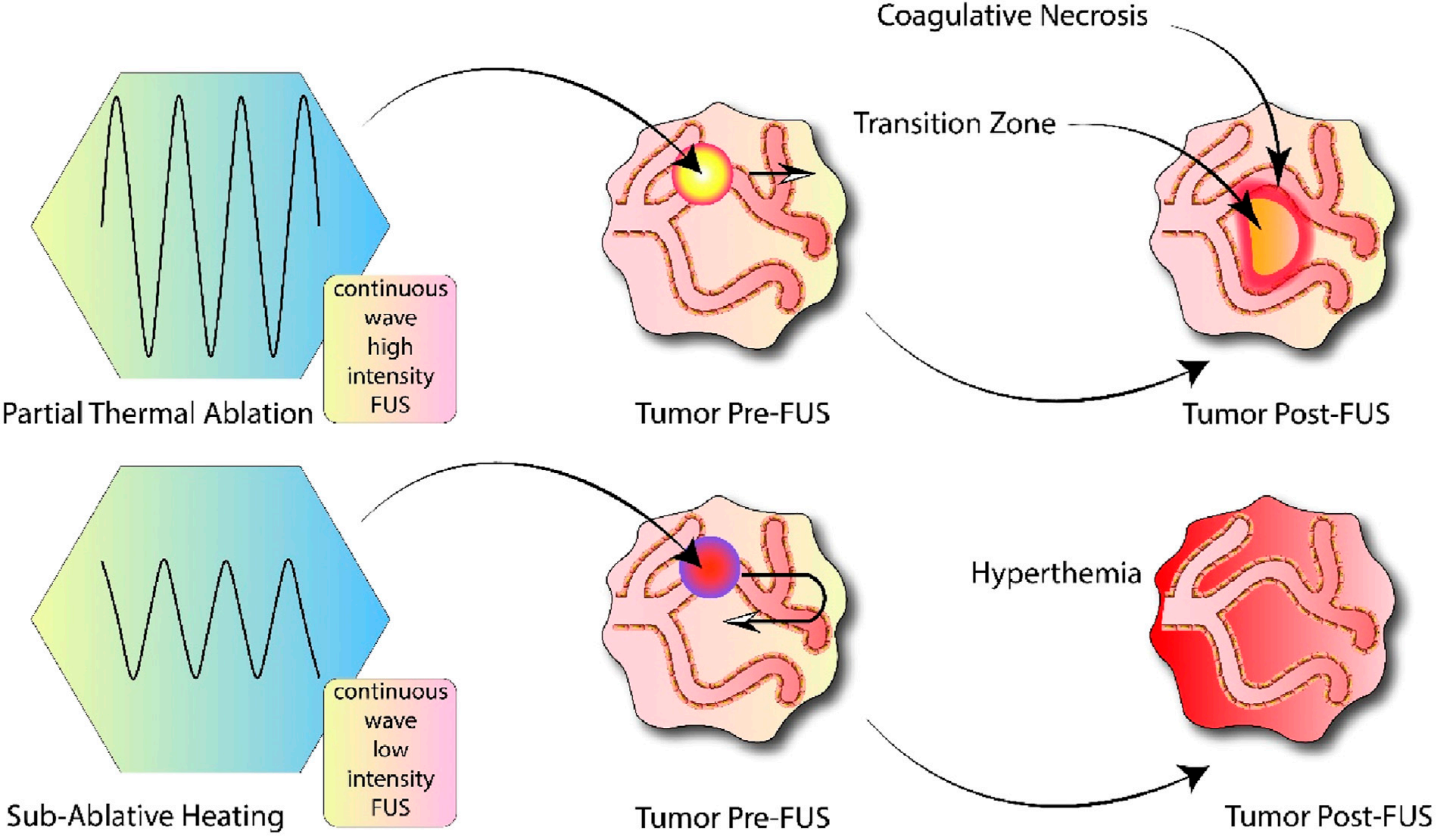
Protocols for cancer immunotherapy utilizing ultrasound-based heat treatment. Left Column: Applying continuous wave focused ultrasound at a high intensity level for partial thermal destruction. By directing the ultrasound beam at high energy levels to a targeted section of the tumor, a region of coagulative necrosis is created. This area is then bordered by a transitional zone leading to the unaffected tumor tissue. Left Column: Targeted tissue warming through the use of gentle, unbroken ultrasound waves. Adjusting the ultrasound focus throughout the entire tumor volume at this energy intensity leads to hyperthermia, but does not immediately obliterate the tumor cells.

Heating caused by ultrasound occurs when biological tissue absorbs ultrasonic energy. In diagnostic ultrasound, the increase in temperature and the likelihood of bioeffects are minimized or almost nonexistent (Fowlkes, 2008) In diagnostic ultrasound, temperature increases and the risk of biological effects are minimized by following specific usage guidelines. This entails following the ALARA principle (ensuring exposure is minimized as much as feasible), keeping average intensity low over time, and making sure that exposure durations are generally short. Therapeutic uses of ultrasonic heating involve either extended periods of exposure with unfocused beams or the application of more intense focused ultrasound compared to diagnostic levels. In physical therapy, applying unfocused heat to highly absorptive tissues like bones or tendons can be regulated to promote improved healing while avoiding damage (Li et al., 2020). Alternatively, heat can be directed by concentrated beams to coagulate tissue, aiming for its ablation (Ter, 2016). Ultrasound-induced heating, which may cause permanent alterations in tissues, depends on the relationship between time and temperature (Sapareto and Dewey, 1984). The impact of ultrasound exposure may result in slight heating, tissue vaporization, coagulative necrosis, or a combination of these effects, depending on the temperature variations (Ter, 2016). Figure 2 provides a simplified overview of how ultrasound impacts thermal, mechanical, and chemical processes.

To clarify the distinct mechanisms by which ultrasound affects biological tissues, Table 1 summarizes the key differences between thermal and mechanical effects, particularly in therapeutic contexts such as HIFU. Understanding these mechanisms is crucial for optimizing safety and maximizing therapeutic benefit in clinical practice.

**TABLE 1:** Distinctions between mechanical and thermal effects of ultrasound.

Effect type	Mechanism	Key features	Clinical examples	Typical applications	Potential risks/Side effects
**Thermal**	Absorption of ultrasound energy by tissues converts acoustic energy into heat.	- Temperature elevation in targeted tissues- Rate and degree depend on frequency, intensity, and duration- Can be focused (as in HIFU) or unfocused	- High-Intensity Focused Ultrasound (HIFU)- Tissue ablation (e.g., tumors, uterine fibroids)	- Ablation of tumors, fibroids, prostate- Hemostasis	- Thermal burns- Unintended necrosis of adjacent tissues- Damage to sensitive structures
**Mechanical**	Direct non-thermal physical effects of ultrasound waves, including: • Cavitation (formation, oscillation, and collapse of microbubbles)• Acoustic streaming• Radiation force	- No significant temperature rise- Microbubble oscillation (stable cavitation)- Bubble collapse (inertial cavitation) can cause shock waves- Shear stress, cell membrane disruption	- Sonoporation- Thrombolysis- Drug/gene delivery (*via* microbubbles or nanodroplets)- Lithotripsy	- Enhanced drug delivery- Clot dissolution- Breaking up kidney stones	- Microvascular injury- Hemorrhage- Cell lysis- Inflammatory responses

**FIGURE 3:**
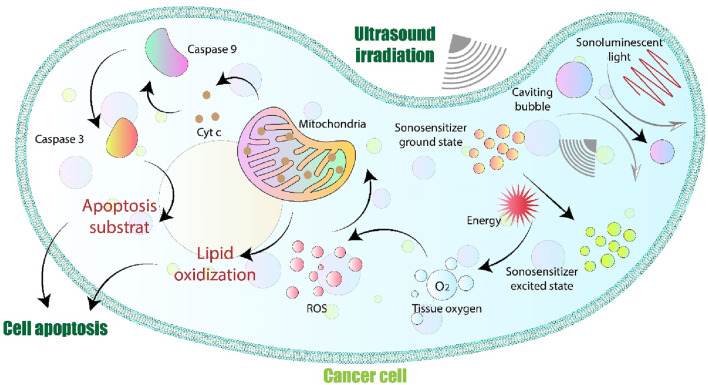
Diagram depicting the different cellular toxicity mechanisms triggered by SDT. Ultrasound-induced cavitation generates tiny gas bubbles within the cell, and it may also lead to the emission of sonoluminescent light. The sonosensitizer is activated and generates reactive oxygen species (ROS), which directly contribute to cellular damage. In particular, cell apoptosis is initiated by harm to the mitochondrial membrane and the discharge of cytochrome c, both of which are enabled by reactive oxygen species (ROS).

Ultrasonic cavitation and gas body activation (refer to Figure 3) are interconnected processes reliant on the low-pressure amplitude generated by ultrasound waves (Rooze et al., 2013). When ultrasound waves move through tissue, they can generate rarefying pressure levels that reach several megaPascals (MPa) (Xu et al., 2009; Zhang et al., 2021). Elevated rarefactional pressure can potentially trigger cavitation in tissues if appropriate nuclei are available, or it may directly cause pulsation in existing gas pockets, such as those found in the lungs and intestines, or when using ultrasound contrast agents (Parsons et al., 2006; Miller, 2007). Local tissue damage, such as cell death and blood vessel hemorrhage, is mainly caused by cavitation and the activation of gas bodies occurring right around the areas where cavitational activity takes place (Arvanitis et al., 2011; Izadifar et al., 2019). Recent studies have expanded our understanding of ultrasound’s biological effects, especially in the context of targeted drug delivery and noninvasive tissue modulation. For instance, the development of microbubble-assisted focused ultrasound has enabled more precise opening of the blood-brain barrier, facilitating delivery of therapeutics for neurological conditions (Burge et al., 2016; Ea et al., 2025). Similarly, high-intensity focused ultrasound (HIFU) is being refined for safer and more effective ablation of deep-seated tumors, with real-time thermal monitoring and adaptive dosing to minimize collateral tissue damage (Shin and Kim, 2024; Ponomarchuk et al., 2022). These innovations not only enhance the clinical utility of ultrasound but also address safety concerns by improving targeting accuracy and reducing undesired bioeffects. Ongoing research aims to optimize parameter selection for different tissue types and disease contexts, suggesting a trend toward more personalized ultrasound therapies (Beiu et al., 2023).

Various ways in which ultrasound might biologically impact organisms involve the direct application of compressional, tensile, and shear forces (Wells et al., 2011; Prado-Costa et al., 2018). Moreover, the secondary effects influenced by the energy of the transmitted ultrasound encompass radiation pressure, forces impacting particles, and the movement of materials known as acoustic streaming. When therapy utilizes high-power or high-intensity ultrasound, multiple mechanisms can simultaneously affect the treatment’s overall biological results. In addition to the immediate physical mechanisms that cause biological effects, there are also indirect physical, biological, and physiological processes that further impact the organism. Instances encompass the narrowing of blood vessels, limited blood circulation (ischemia), fluid leakage from blood vessels (extravasation), tissue harm resulting from the return of blood supply (reperfusion injury), and reactions from the immune system (Silberstein et al., 2008; Hundt et al., 2007; Alves et al., 2009). At times, the indirect consequences can be more significant than the immediate impact of the ultrasound.

## 3 Ultrasound molecular applications

### 3.1 Induction of cell death by ultrasound

Since ultrasound first causes distortions in the cell membrane through mechanical impact, the external kinetic energy can penetrate deeply within the cell through intricate mechanotransduction pathways, altering the cell’s function (Furusawa et al., 2014). Mechanotransduction is the process by which cells convert mechanical stimuli into biochemical signals, allowing them to respond to changes in their environment (Ingber, 2006). This pathway involves the detection of mechanical forces, such as stretch, shear stress, or pressure, through mechanoreceptors on the cell surface (Di et al., 2023). These receptors include integrins, ion channels, and cell adhesion molecules. Mechanical forces applied by ultrasound are transduced *via* cell surface receptors—such as integrins and ion channels—into intracellular signaling cascades that regulate gene expression and cell behavior (Di et al., 2023; Ohashi et al., 2024; Xie et al., 2023; Gargalionis et al., 2024; Di-Luoffo et al., 2021; Li et al., 2023; Wang et al., 2023). These pathways can regulate cellular functions such as gene expression, cell growth, differentiation, and migration (Ohashi et al., 2024; Xie et al., 2023; Gargalionis et al., 2024; Di-Luoffo et al., 2021; Li et al., 2023; Wang et al., 2023). In tissues like muscle, bone, and endothelial cells, mechanotransduction is critical for processes such as tissue remodeling, bone formation, and vascular health (Wang, 2017; Ingber, 2003; Bakhshandeh et al., 2023). Abnormalities in mechanotransduction pathways are implicated in various diseases, including cancer, fibrosis, and cardiovascular disorders (Lammerding et al., 2004; Garoffolo and Pesce, 2019; Kuehlmann et al., 2020; Chin et al., 2016). So far, many studies have documented a range of cellular changes induced by ultrasound, such as reduced cell viability (Lai et al., 2006), disruption of cell membrane potential (Tran et al., 2008; Tran et al., 2007), changes in calcium signaling (Fan et al., 2010; Park et al., 2010), generation of reactive oxygen molecules (Hassan et al., 2010a; Okada et al., 2009) or the generation of shear force (Park et al., 2011; Collis et al., 2010). Mechanisms established by the US, among others, are crucial in affecting the biological changes that significantly alter the intracellular environment as a result of drug-induced sonoporation, ultimately determining the therapeutic results.

An ultrasound creates a series of pressure changes by transmitting sound waves through tissue. When these waves encounter reflective surfaces, such as tissue interfaces or structures with different acoustic impedances, they can form a spatial standing wave pattern (Zhu et al., 2003; Scorer, 1967). Standing waves occur when incident and reflected waves interfere with each other, creating regions of constructive and destructive interference, known as pressure nodes and antinodes (Prants, 2009; Sanlı et al., 2014). This can lead to non-uniform energy deposition, which may cause localized heating or other unintended effects.

The occurrence of standing waves in ultrasound is particularly relevant in therapeutic applications, such as HIFU, where precise energy delivery is critical (Song et al., 2010; Song et al., 2011). While standing waves are relatively common in areas where ultrasound waves reflect off hard surfaces or large tissue interfaces, their impact can be mitigated by using techniques such as real-time monitoring of the ultrasound field, beam steering, or changing the frequency of the ultrasound waves (Casper et al., 2013; Xiao et al., 2024). Solutions like phased-array transducers, which allow for dynamic beam focusing and steering, are commonly used to minimize the formation of standing waves and ensure more even energy distribution (Bosma et al., 2022; Ca et al., 2019; Bawart et al., 2020). Addressing standing wave formation is crucial in therapeutic ultrasound to prevent tissue damage and improve treatment precision.

Overall, the biological effects are greatly affected by the specific ultrasound parameters utilized, the distance between the energy source and the target cells, the transducer or any components causing a standing wave, and the presence of acoustically active microbubbles (Hassan et al., 2010b; Nyborg et al., 1985). Multiple investigations employing scanning electron microscopy and atomic force microscopy have verified that physical pores develop in the cell membrane after being exposed to ultrasound (Zhao et al., 2008; Taniyama et al., 2002; Mehier-Humbert et al., 2005; Duvsha et al., 2006). Reversible sonoporation (RS) enhances the absorption of drugs by cells without resulting in cell death. In contrast, irreversible sonoporation (IRS) is thought to be deadly, often causing rapid cell necrosis (Lentacker et al., 2014; Fan et al., 2014; Qin et al., 2018; van Rooij et al., 2016). Additionally, these phenomena become significantly more pronounced when gas-filled microbubbles are present. When sound waves come into contact with microbubbles, the interaction between ultrasound and the microbubbles creates significantly stronger forces, enhancing the bioeffects initiated by the ultrasound (Schlicher et al., 2006; Deng et al., 2004; Zhong et al., 2011).

In the past few years, research on ultrasound-mediated cell death and sonoporation has moved beyond fundamental mechanistic studies, with a focus on translating these findings into clinical and preclinical settings. For example, recent studies have explored the use of ultrasound-induced sonoporation for targeted gene and drug delivery in cancer therapy, with promising results in animal models and early-phase clinical trials (Krut et al., 2022; Entzian and Aigner, 2021; Sitta and Howard, 2021). Innovations such as acoustic cluster therapy and smart microbubble formulations have demonstrated enhanced specificity and controllability in delivering therapeutic agents (Edwards et al., 2023; Del Campo Fonseca, 2024; Tsirkin et al., 2021). There is growing interest in using ultrasound in combination with immunotherapies to potentiate immune cell infiltration and enhance antitumor responses (Sun S. et al., 2022; Yang et al., 2024). Furthermore, high-resolution real-time imaging modalities now enable researchers to visualize membrane pore formation and cytoskeletal changes dynamically during and after ultrasound exposure (Wen et al., 2023; Jia et al., 2022). Collectively, these advances are shaping a new generation of ultrasound-based molecular therapies, while also revealing important challenges regarding selectivity, safety, and optimal parameter settings for clinical applications (Przystupski and Ussowicz, 2022; Rich et al., 2022).

Previous studies have shown that sonoporation can occur in two ways: using microbubbles, which is called bubble-based sonoporation (BBS), or without the use of microbubbles, known as non-bubble-based sonoporation (NBBS). While each mechanism leads to membrane permeabilization, the underlying biophysical processes that create pores are notably distinct. When sound waves pass through fluid-filled cells, the cells undergo shear forces due to the concurrent effects of acoustic streaming and the acoustic radiation force. This setup could weaken the cell membrane, resulting in the development of holes (Rich et al., 2022; Liu et al., 2017; Ahmed et al., 2016). These phenomena were utilized to create a range of mechanisms aimed at facilitating NBBS. This involves using traveling sound waves to eject cells through nozzle openings (Zarnitsyn VG. et al., 2008), exposing cells to stationary sound waves within resonators that measure a quarter of the wavelength (Carugo et al., 2011; Carugo et al., 2014), or using Lamb waves with cells grown on delicate substrates (Gedge and Hill, 2012; Friend and Yeo, 2011; Huang et al., 2021). Some researchers also utilized bulk acoustic waves traveling across the transducer to activate NBBS. Multiple options emerge when the target cells are located near the US origin. In this scenario, concentrated areas of strong acoustic energy can be created by focusing bulk acoustic waves into a small region (Kim et al., 2021; Yoon et al., 2017; Yoon et al., 2016), As another method, using high-frequency bulk acoustic waves can induce acoustic streaming, which leads to the formation of pores in the cell membrane (Kamenac et al., 2021; Ramesan et al., 2021). In the United States, ultrasound contrast agents frequently comprise microbubbles (MBs) with a gas-filled center encased by a stabilizing layer of lipids, polymers, or albumin. Due to their size, usually between 1 and 8 μm in diameter, they can easily pass through capillaries throughout the human body (Polat et al., 2011; Goldberg et al., 1994; Kooiman et al., 2014). Utilizing microbubbles as cavitation nuclei reduces the cavitation threshold by enhancing sound energy absorption, which in turn amplifies the effects produced by ultrasound (Yang et al., 2020; Chen W-S. et al., 2003; Chen W- et al., 2003). When subjected to ultrasound, microbubbles exhibit various behaviors leading to acoustic cavitation (Shamout et al., 2015; Yu et al., 2015). Rapid changes in volume during acoustic cavitation cause various mechanical, chemical, and thermal effects that significantly alter the surrounding environment, ultimately resulting in bubble-induced sonoporation (Kooiman et al., 2020). It is important to recognize that different forms of acoustic cavitation can occur depending on the intensity of the ultrasound, known as the mechanical index. To calculate the mechanical index (MI), you need to divide the peak negative pressure in megapascals (MPa) by the square root of the acoustic wave’s central frequency in megahertz (MHz) (Snipstad et al., 2018). The effect of ultrasound on cellular biology is significantly determined by the mechanical index. When the mechanical index drops below 0.2, microbubble volumes continuously fluctuate, leading to pressure and tension on the cell membrane, ultimately causing it to break (Doinikov and Bouakaz, 2010). This method is referred to as stable cavitation (Postema et al., 2012).

The relationship between ultrasound parameters and biological outcomes is critical for both safety and therapeutic efficacy. Table 2 summarizes key ultrasound parameters, their typical values, and the main biological or therapeutic effects observed at different thresholds, with emphasis on cavitation phenomena and tissue response.

**TABLE 2:** Key ultrasound parameters and associated biological/therapeutic effects.

Parameter	Typical range/Threshold	Associated biological/Therapeutic effects	Clinical/Experimental contexts
Frequency (MHz)	0.5–3 (therapy); 2–15 (diagnostic)	Lower freq = deeper penetration, higher cavitation potential; higher freq = superficial, less cavitation	HIFU, LIPUS, diagnostic imaging
Mechanical Index (MI)	<0.2	Stable cavitation; microbubble oscillation without collapse	Drug/gene delivery, imaging contrast agents
	0.2–0.6	Increased membrane permeability, reversible sonoporation, some risk of cell lysis	Sonoporation, targeted delivery
	>0.6	Inertial (transient) cavitation: microbubble collapse, jetting, shockwave, possible cell/tissue damage	HIFU ablation, lithotripsy, thrombolysis
Intensity (W/cm^2^)	<0.1 (diagnostic); 0.5–10 (therapy)	<0.1: safe for imaging; >1: possible tissue heating or damage	Diagnostic US, therapeutic ablation
Exposure Duration	ms – minutes	Short pulses = less heating; prolonged = thermal accumulation	Imaging vs therapeutic protocols
Duty Cycle	0.1%–100% (pulsed vs continuous)	Low duty cycle = reduced thermal effect; continuous = more heating	LIPUS, HIFU, physiotherapy

**FIGURE 4:**
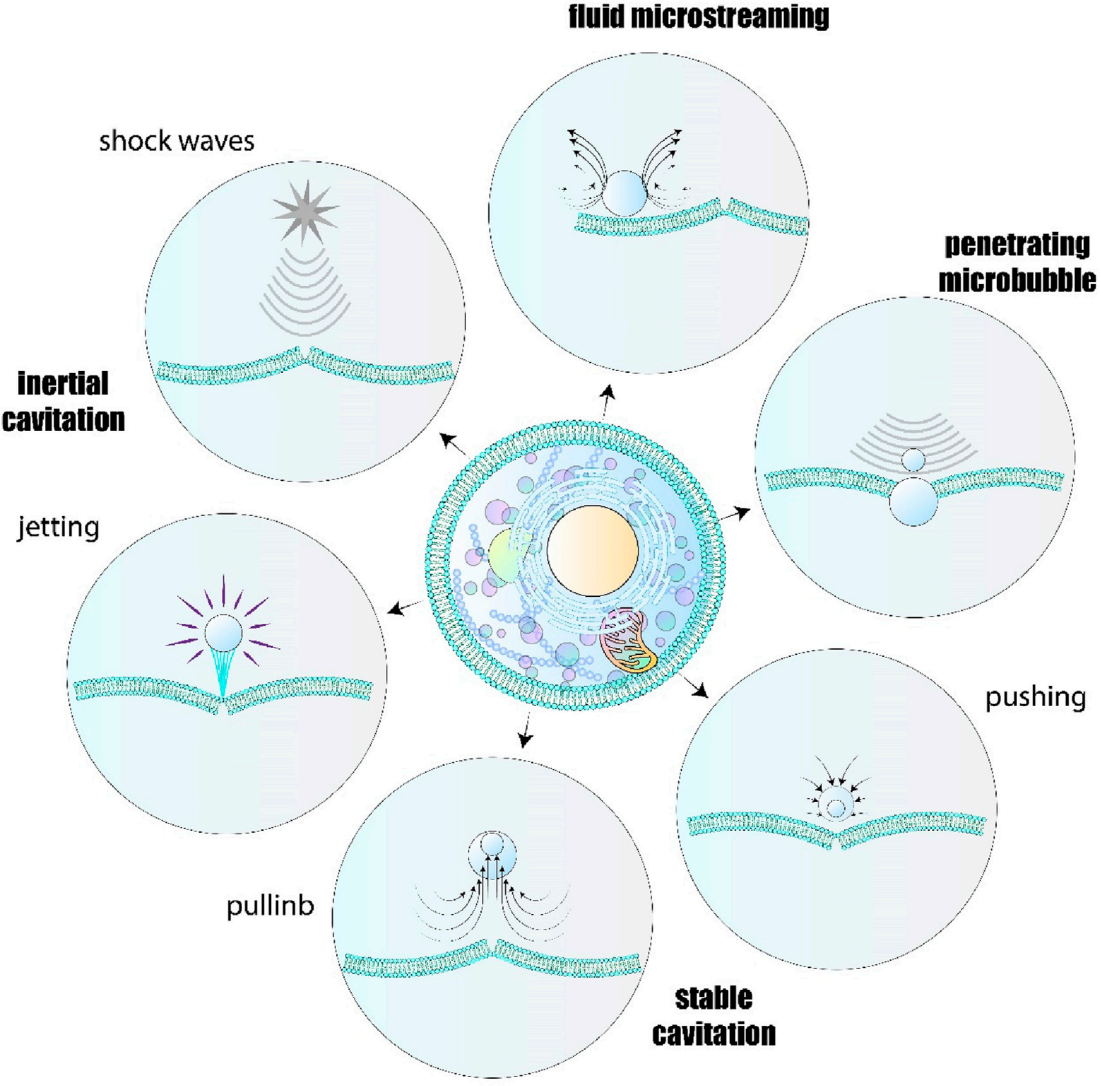
Illustrative depiction of the physical processes involved in sonoporation. Ultrasound could induce sonoportion *via* different mechanisms including pulling, pushing, fluid microstreaming, penetrating microbubble, jetting, and production of shockwave. Based on the physico-chemical properties of membrane and bubbles, the main mechanism could be different.

Understanding that microbubbles attaching to the cell membrane can produce oscillations that result in fluid motion is crucial, as this can create a local shear stress capable of rupturing the cell membrane. This occurrence is known as microstreaming (Escoffre et al., 2020). In this case, microbubbles could potentially traverse the lipid bilayer, resulting in membrane permeabilization. When the MI value surpasses 0.2, the intensity of the acoustic pressure grows, resulting in a notable rise in the kinetic energy released. In instances like this, the MBs experience intense oscillations, resulting in a phenomenon known as inertial cavitation. This happens when they fall apart and break down, creating shock waves or tiny jets that penetrate the lipid bilayer (Zhou, 2011; Ohl and Wolfrum, 2003; Junge et al., 2003). Figure 4 illustrates the mechanical effects produced by microbubbles. When ultrasound is applied, it causes repeated cycles of compression and expansion at varying pressures among the molecules, significantly increasing the medium’s temperature due to the energy transferred. The rise in temperature leads to the heat-induced effects seen in BBS. The rise in temperature is directly connected to the original energy converted into the sound wave, leading to overheating in specific areas (Kooiman et al., 2020). This process is utilized in HIFU-ablation treatment for cancer therapy (Zhou, 2011). Furthermore, it was asserted that hyperthermia caused by ultrasound in the US could enhance drug absorption by altering membrane permeability, particularly in cells resistant to multiple drugs (Liu et al., 2001). Given that BBS involves significant mechanical and thermal effects, both factors can play a role in the chemical reactions taking place at BBS. A significant rise in gas pressure or a rapid temperature increase when microbubbles collapse can result in the creation of reactive oxygen species (ROS) and the release of electromagnetic waves, a phenomenon known as sonoluminescence. Interestingly, both sonoluminescence and reactive oxygen species (ROS) have the ability to modify drug resistance (Trachootham et al., 2009; Ros et al., 2004; Be et al., 2019).

### 3.2 Cell membrane modifications

Ultrasound exposure causes openings to develop in the cell membrane. Numerous scientists have attempted to ascertain the dimensions of these pores, proposing that they are circular with a specific diameter. Their study was based on the premise that passive diffusion serves as the main mechanism by which ultrasound enhances the entry of molecules into cells. SEM analysis offered clear proof of membrane permeabilization, revealing irregularly shaped pores ranging in size from 100 nm to several micrometers (Mehier-Humbert et al., 2005; Fan et al., 2014). A range of studies using various cell models and techniques have found that sonoporation-induced membrane pores typically range from approximately 50–2,500 nm, with smaller pores more likely to reseal quickly. The efficiency of molecular uptake correlates with pore size and exposure conditions (Zarnitsyn V. et al., 2008; Zhou et al., 2009; Fan et al., 2012). Multiple microscopy studies have shown that ultrasound-induced sonoporation generates membrane pores ranging from tens to thousands of nanometers, often with increased surface roughness and bleb formation (Sanlı et al., 2014; Song et al., 2010; Song et al., 2011; Bawart et al., 2020; Chen W-S. et al., 2003; Chen W- et al., 2003). The extent of these changes correlates with acoustic exposure and pressure, though reported pore sizes can vary due to methodological differences in imaging and sample preparation (Zhao et al., 2008; Taniyama et al., 2002; Mehier-Humbert et al., 2005; Schlicher et al., 2006; Duvshani-Eshet et al., 2006; Yang et al., 2008).

Some researchers have also explained how the diameter of bubbles and the distance between bubbles and cells relate to the extent of permeabilization caused by ultrasound. As an illustration, Qin and colleagues explored the immediate response of individual cells to sonoporation when initiated by the vaporization of acoustic droplets. They contended that substantial ADV bubbles resulted in permanent sonoporation only when located close to the cell, as those positioned far from the cell membrane did not succeed in initiating sonoporation. This finding suggested that the final effect of sonoporation was affected by both the size of the microbubble and how close it was to the cell. A higher likelihood of irreversible sonoporation occurred when the bubble was nearer to the cell and had a larger diameter (Qin et al., 2018). In a study conducted by Hu and colleagues (Hu et al., 2013) It was discovered that the ability of sonoporation to reverse was dependent on the size of the pores formed; pores with a surface area smaller than 30 μm^2^ sealed within a minute following ultrasound treatment, whereas those larger than 100 μm^2^ stayed open for as long as 30 min. Additionally, post-sonoporation cell viability was influenced by how quickly the membrane resealed: cells remained viable after ultrasound treatment only if their pores closed within 1 minute (Hu et al., 2013; van Wamel et al., 2006; Zhou et al., 2012). It is crucial to understand that the degree of membrane damage in BBS is affected by the sonoporation technique. When microbubbles undergo inertial cavitation, they produce tiny openings that can measure up to several hundred nanometers. Conversely, stable microbubble cavitation results in the creation of larger openings, which can range in size from several hundred nanometers to a few micrometers (Forbes et al., 2008; Wang et al., 2018).

The degree of effectiveness of sonoporation can differ among sonoporated cells at a specific moment because the ultrasound-induced process of making the cell membrane permeable can be affected by various factors simultaneously. These factors include the bubble’s size, its proximity to the cell, and the acoustic wave’s energy. Multiple studies have furnished evidence supporting the variation in particle uptake. Guzmán and his team proposed that the varying levels of ultrasound and cavitation experienced by different cells explained this occurrence. Nonetheless, they were unable to observe a complete spectrum of uptake intensities in the sonoporated cells. Additionally, De Cock and his team identified only two separate cell groups that showed differing levels of molecule uptake. Through the use of confocal microscopy to examine different subgroups of sonoporated cells, researchers discovered that these cells demonstrated multiple uptake processes. Specifically, at lower levels of uptake intensity, endocytic uptake was noticed, whereas at higher uptake intensity, cell membrane permeabilization was observed. They also observed that raising the acoustic pressure led to an increased number of sonoporated cells with significant uptake. Consequently, they determined that decreasing the pressure enhances endocytosis, while increasing the pressure fosters pore creation, suggesting that modifying the acoustic wave pressure can influence the uptake method (De Cock et al., 2015; Gu et al., 2001).

**FIGURE 5:**
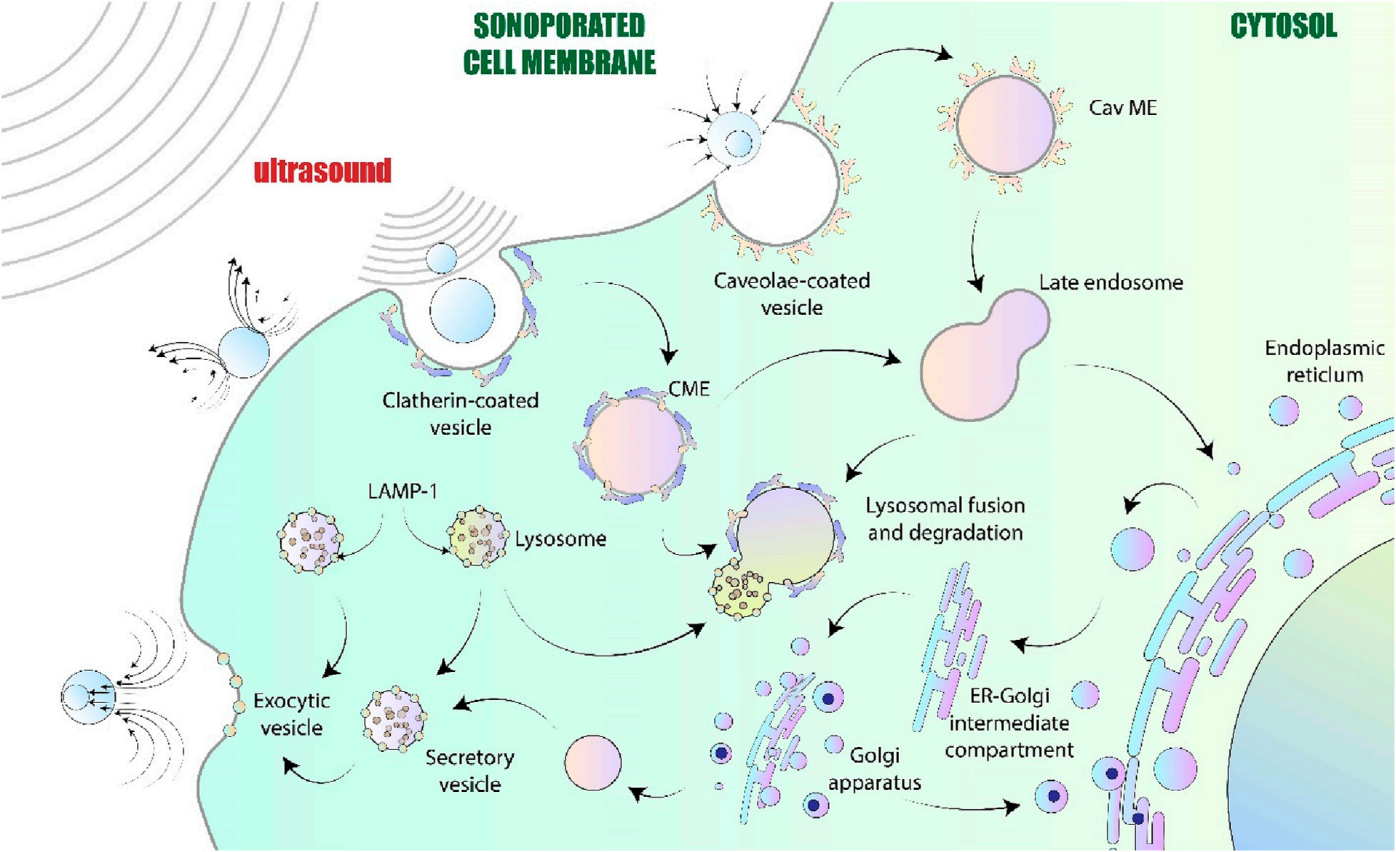
Membrane-resealing mechanisms triggered by US.

To promote cell survival and limit the movement of different substances through the membrane, the openings formed in the cell membrane by ultrasound should be closed before they naturally seal up. Fixing membranes is essential to stop ions from building up inside the cell, which can disturb its internal balance. Previous research suggests that sonoporation may activate two mechanisms for membrane resealing (see Figure 5): membrane repair initiated by endocytosis and vesicular patching associated with exocytosis (Leow et al., 2015). The mechanical influence applied by the US can lead to deformation of cell membranes by either pushing microbubbles against them or through stable cavitation, which creates microstreaming in the surrounding fluid. These occurrences alter the cell membrane’s tension without compromising the plasma membrane’s structure, creating shear stress that collectively results in the rearrangement of cytoskeletal fibers. Mechanosensors, like integrins or stretch-activated ion channels, can detect these mechanical forces and convert them into signals that alter cell activities (Hauser et al., 2009). The processes of rebuilding, called exocytosis, and removal, known as endocytosis, are said to restore the cell membrane’s initial tension and structural integrity (Lentacker et al., 2014). It is widely thought that the primary mechanism for sealing small openings formed by oscillating microbubbles is endocytosis (Qin et al., 2018). Conversely, large membrane pores caused by collapsing microbubbles are repaired through exocytosis and lysosomal patches (Yang et al., 2008; Schlicher et al., 2010). Additionally, only pores with a size less than 0.2 µm are capable of being effectively resealed (McNeil and Steinhardt, 2003). This accounts for the inconsistent findings from various groups, with some reporting only US-induced exocytosis and others reporting endocytosis in experiments related to drug uptake.

After undergoing US treatment (Ogawa et al., 2001; Chen et al., 2013), the cells exhibited a reduction in size, and their surfaces appeared flatter and smoother (Duvshani-Eshet and Machluf, 2005). The examination of sonoporated DU-145 prostate cancer cells through scanning electron microscopy (SEM) and confocal microscopy showed cell damage, which was marked by circular projections known as ‘balloons' and ‘blister' blebs. The extensions in the shape of balloons held small lipid vesicles produced by the endoplasmic reticulum, while the blister-like extensions consisted of lipids that fused with the cell membrane and contained a clear fluid. Schlicher and colleagues (Schlicher et al., 2010) noted a variety of occurrences such as several types of membrane protrusions, the expulsion of the nucleus, the creation of structures surrounding the nucleus, and even the breakdown of cells. Zeghimi and colleagues employed TEM and SEM microscopy methods to study how the plasma membrane of U-87 MG cells changes over time. They noticed a large amount of exposed holes and tears in the membrane, almost all of which were fixed within an hour (Zeghimi et al., 2015). Nevertheless, certain structural changes, including the formation of pit-like characteristics and increased membrane roughness, persisted up to 24 h after ultrasound treatment with opsonin microbubbles (Taniyama et al., 2002; Duvshani-Eshet et al., 2006). Cell membrane blebs were among the morphological changes seen following sonoporation. Qin and colleagues noted the appearance of blebs 90 s post-exposure, which subsequently grew and led to the formation of additional blebs. These structures were detected in cells that underwent both reversible and irreversible sonoporation (Qin et al., 2018). Additionally, Leow and colleagues (Leow et al., 2015) observed secondary blebs forming in areas not directly affected by sonoporation. Blebs are believed to develop as a defensive mechanism to alleviate heightened hydrostatic pressure and restore equilibrium, caused by ultrasound-induced disruption of the actin cytoskeleton (Leow et al., 2015; Chen et al., 2014). Honda and colleagues additionally identified apoptotic cells 6 hours following sonication. The nucleus and cell body notably shrank, accompanied by chromatin condensation and fragmentation of the nucleus. However, only some dying cells exhibited cytoplasmic swelling and an enlarged endoplasmic reticulum. In addition, vacuolar formations were observed and identified as autophagic vacuoles and secondary lysosomes (Honda et al., 2004). Tachibana et al. (Tachibana et al., 1999) noticed a decrease in the quantity of microvilli and membranous laminar ruffles in HL-60 cells following ultrasound exposure. In the subsequent years, confocal imaging revealed that lipid concentrations rose in specific damaged regions and progressively spread outwards. Furthermore, linear arrangements of lipids were observed on the cell membranes of HL-60 cells exposed to ultrasound. The researchers demonstrated that the newly formed lipids originated from within the cell (Hu et al., 2019). In addition, after undergoing sonoporation, sonoporated MCF-7 cells lacked microvilli, leading to a smooth cell surface. Many of these cells appeared round and shrunken. The damage to the membranes was notably more extensive in MCF-7/ADR cells that were resistant to doxorubicin (Jia et al., 2015). Ovarian cancer cells exposed to US displayed enlarged mitochondria and vacuoles within the cytoplasm (Yu et al., 2004).

### 3.3 Cytoskelton

Ultrasound-induced sonoporation leads to rapid and reversible changes in the cytoskeleton, including disruption of F-actin filaments, cytoskeletal rearrangement, and altered cell adhesion and migration (Escoffre et al., 2020; Duvshani-Eshet et al., 2006; Zhou et al., 2012; Forbes et al.; Wang et al., 2018; De Cock et al., 2015; Gu et al., 2001; Leow et al., 2015). These changes are thought to facilitate membrane repair and may influence cellular signaling and mechanical properties. However, the specific pathways involved and their long-term impact on cellular function require further investigation. (Wang et al., 2018; Chen et al., 2014; Gourlay and Ayscough, 2005; Revenu et al., 2004; Juffermans et al., 2009; Fan et al., 2013; Helfield et al., 2016). Despite considerable progress in elucidating the mechanisms of ultrasound-induced cellular effects, future studies will be critical for optimizing parameter selection and integrating real-time monitoring to enhance both safety and therapeutic efficacy in clinical applications.

## 4 Ultrasound in molecular imaging

### 4.1 An overview of using ultrasound in molecular imaging

The purpose of various molecular ultrasound imaging and quantification methods is to distinguish the signal generated by microbubbles that are bound to specific molecular targets (the molecular signal) from the signals of unbound microbubbles, which do not exhibit molecular-specific binding, and from the background tissue signal. Targeted molecular imaging allows for the visualization of specific biological processes at a molecular level, which provides much greater sensitivity and specificity compared to traditional ultrasound (Deffieux et al., 2021; Jiang et al., 2023). This technique can be used to detect early-stage diseases, identify tumors, assess blood flow, or monitor the effectiveness of therapies by targeting specific biomarkers associated with disease (Eisenbrey et al., 2021; Laghi et al., 2021). In cases where precision is crucial—such as in oncology, cardiology, or neurology—these microbubbles can significantly improve the diagnostic accuracy, aiding in earlier intervention and better patient outcomes (Laghi et al., 2021; Wang et al., 2021). While the simplicity and affordability of traditional ultrasound are valuable, targeted microbubbles offer a more sophisticated approach when the clinical need for detailed, molecular-level insights outweighs the cost and complexity.

In numerous early ultrasound systems, untargeted microbubbles were rendered visible by using high-intensity ultrasound pulses to disintegrate them. This method is often called Loss of Correlation (LOC). When a microbubble bursts, it generates several acoustic signals across different imaging pulses, leading to a notable decorrelation detected during the analysis of Power Doppler signals (Becher and Burns, 2012). Although this method is compatible with current Power Doppler imaging systems, it has limitations in distinguishing between contrast agents (microbubbles) and surrounding tissues (Correas et al., 2006; Quaia, 2007; Sridharan et al., 2021). This is because both tissues and contrast agents can produce similar Doppler signals, making it challenging to differentiate the specific signal originating from the microbubbles, which are designed to enhance imaging (Sridharan et al., 2021). As a result, the method may not provide the level of specificity needed for accurate molecular imaging, where the goal is to isolate signals from targeted microbubbles bound to specific biomarkers, rather than from general tissue or untargeted microbubbles. Furthermore, molecular ultrasound imaging is inappropriate due to the fact that the required microbubble signal is rapidly disrupted during the imaging procedure. Consequently, innovative imaging methods have been created that take advantage of the distinct characteristics of microbubbles. Due to their high compressibility and the adaptable structure of their shells, microbubbles undergo nonlinear oscillations when subjected to an ultrasound field (Leighton, 2012). As a result, the ultrasound waves disperse in a nonlinear fashion, generating harmonics at multiples of the main frequency, which can include both higher-order harmonics, such as second and third harmonics, as well as subharmonics and fractional harmonics (e.g., 3/2 of the main frequency). This includes twice the frequency (the second harmonic) and further multiples like the third, fourth harmonics, and more (Bouakaz et al., 2002; De Jong et al., 1994), or at a fraction of the central frequency, specifically one-half (subharmonic) (Forsberg et al., 2000). Consequently, most ultrasound imaging techniques focus on detecting this nonlinear energy generated by microbubbles, enabling them to distinguish the imaging signals from the microbubbles from those coming from the tissue (Deng and Lizzi, 2002). As acoustic pressure rises, tissue exhibits a nonlinear response to ultrasound (Hamilton and Blackstock, 2024), regularly generating a non-linear signal distinct from the tissue background. Using low acoustic pressures, generally less than 500 kPa, significantly reduces signals from the surrounding tissue, enhancing the contrast between the ultrasound image and the tissue. Harmonic Imaging (Chang et al., 1995; Burns et al., 1994) was developed to visualize microbubbles non-linearly, overcoming the natural limitations present in contrast-enhanced ultrasound using LOC methods. The ultrasound system’s analog electronics utilized frequency-based filters to retain particular nonlinear frequency components, referred to as harmonics. These implementations enhanced detection relative to LOC, yet they were constrained by a limited signal bandwidth because of the way analog harmonic filters were designed. This is because frequency-based filters are unable to differentiate between the linear and nonlinear parts of a signal when both share the same frequency. Limiting the frequency range used in imaging helps reduce signal overlap, but this comes at the cost of decreased resolution along the axial direction.

In the last few years, molecular ultrasound imaging has rapidly advanced from primarily preclinical proof-of-concept work to early-phase clinical translation. Novel microbubble designs—such as dual-targeted, drug-loaded, and stimuli-responsive agents—are being explored to increase specificity and sensitivity for disease biomarkers (Park et al., 2024; Ma et al., 2022; Kida and Tachibana, 2023). Integration of artificial intelligence and machine learning algorithms into ultrasound image analysis has also enabled automated, more quantitative, and reproducible assessment of molecular signals (Fu et al., 2024; Li H. et al., 2024; Saba et al., 2025). Despite this progress, key challenges remain, including large-scale agent manufacturing, regulatory approval, and robust validation of quantification protocols for routine clinical use. Nevertheless, these ongoing innovations signal a major shift toward personalized, image-guided therapy and monitoring using molecular ultrasound technologies.

The most frequently used two-pulse sequences are Pulse Inversion (PI) and Amplitude Modulation (AM), also known as Power Modulation. Various multi-pulse methods are generally founded on the principles of PI, AM, or a combination of both (PIAM). The standard setup uses two pulses, but it can be extended to include several sequential pulse pairs (Eckersley et al., 2005; Phillips, 2001). Overall, increasing the number of pulses in a sequence improves the signal-to-noise ratio, thereby enhancing sensitivity, although it does not directly reduce tissue motion. However, this results in a reduced frame rate, which in turn lowers the temporal resolution (Simpson et al., 2001; Averkiou, 2001).

### 4.2 Ultrasound in tumor angiogenesis

Tumor angiogenesis refers to the formation and recruitment of new blood vessels from the surrounding tissue of the host. During this process, a range of molecular markers are prominently expressed on the endothelial cells lining the tumor’s blood vessels (Hanahan and Weinberg, 2000; Folkman, 2006). Microbubbles designed to attach to specific molecular markers associated with angiogenesis, including vascular endothelial growth factor receptor type 2 (VEGFR2), αvβ3 integrin, or endoglin, have been used to monitor tumor angiogenesis through molecular ultrasound imaging methods. In the study carried out by Lee and his team (Lee et al., 2008), a clear positive relationship was found between the ultrasound imaging signals from living subjects and the true levels of VEGFR2 expression on endothelial cells in subcutaneous breast cancer tumors. Due to the fact that the expression of angiogenesis markers differs depending on the type of tumor and the stage of its development, another team has focused on this area (Willmann et al., 2008) investigated the application of microbubbles engineered to simultaneously target VEGFR2 and αvβ3 integrin, aiming to enhance imaging signals in human ovarian cancer xenografts grown under the skin in mice. The *in vivo* imaging results displayed a substantial improvement in signal when dual-targeted microbubbles were employed, compared to using separately targeted microbubbles or a mixture of two different singly-targeted microbubbles. This implies that utilizing contrast microbubbles directed at various targets might enhance the detection of tumor blood vessel formation. This could be vital for molecular ultrasound imaging in the early identification of tumors during cancer progression, as unique markers appear at various stages.

Recent studies have demonstrated that microbubbles conjugated with multiple ligands can not only improve detection sensitivity but also enable dynamic imaging of angiogenic responses to emerging immunotherapies and small-molecule inhibitors (Zhong et al., 2023; Jugniot et al., 2021). Machine-learning-driven analysis of these signals is providing more nuanced characterization of tumor microenvironment changes during therapy in preclinical and pilot clinical studies (Kierski, 2022).

Furthermore, three independent studies have demonstrated that molecular ultrasound enables continuous monitoring of anti-angiogenic treatment in human cancer xenograft tumors implanted in mice. Subcutaneously and orthotopically implanted pancreatic tumors were treated using anti-VEGF monoclonal antibodies along with or without gemcitabine. For monitoring purposes, continuous molecular imaging was carried out utilizing microbubbles that were specifically engineered to bind with VEGFR2, the VEGF-VEGFR complex, or endoglin (Korpanty et al., 2007). In tumors treated with anti-angiogenic or cytotoxic therapies, the imaging signal from targeted microbubbles diminished. This reduction was associated with the expression levels of the target and the density of microvessels. In untreated subcutaneous human squamous cell xenografts in mice, the levels of VEGFR2 and αvβ3 integrin were elevated. However, after administering a matrix metalloproteinase inhibitor, both markers demonstrated a reduction (Palmowski et al., 2008). Pysz et al. (Pysz et al., 2010) We examined the molecular ultrasound imaging signals in mouse models simulating human colon cancer, both with and without the application of anti-angiogenic therapy. This was achieved using ultrasound technology and a new human VEGFR2-targeted microbubble, which holds promise for clinical use. Just 1 day after starting anti-angiogenic treatment, molecular ultrasound revealed a notable reduction in imaging signals in the treated mice compared to the untreated ones, even though there was no change in tumor size at this initial phase of therapy. The study showed that molecular ultrasound has the capability to assess the efficacy of anti-angiogenic therapy at an early phase, prior to when visible morphological or anatomical alterations become evident in tumors.

### 4.3 Ultrasound for imaging of inflammatory processes

**FIGURE 6:**
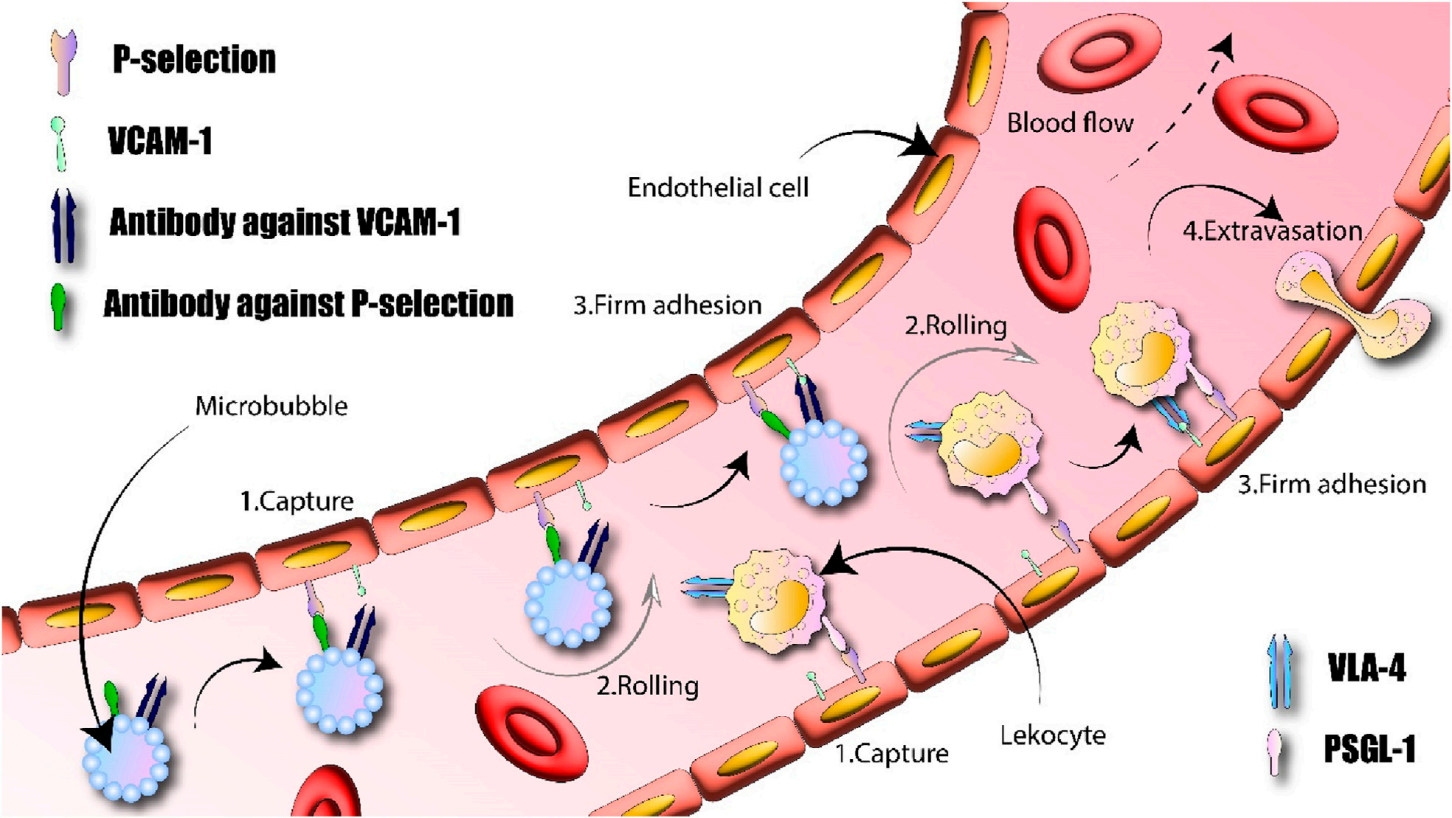
It is feasible to design a microbubble that targets two specific areas and imitates the actions of leukocytes within the body. 1) They are initially captured and 2) roll along blood vessels due to temporary interactions between selectin proteins (for example, P-selectin) and their corresponding molecules (such as P-selectin glycoprotein ligand-1 - PSGL-1); 3) They then firmly attach to endothelial cells through strong bonds between cell adhesion molecules (like VCAM1) and integrins (such as Very Late Antigen-4 - VLA-4); and 4) Finally, they undergo the process of extravasation.

Inflammation is a typical bodily reaction involved in a variety of diseases. Consequently, a noninvasive imaging technique that can evaluate inflammation at the molecular level in the body could be advantageous for early diagnosis and tracking the effectiveness of treatment. A typical feature of inflammation is the activation of white blood cells in the bloodstream, causing them to move into tissues outside the blood vessels. The interaction between adhesion molecules on white blood cells and different receptors on the surface of endothelial cells facilitates the movement and distribution of these white blood cells (Lindner et al., 2000; Kunkel and Butcher, 2003). Molecules like E-selectin and P-selectin play a crucial role in the initial phase of white blood cell capture, facilitating their movement along the interior lining of blood vessels. As depicted in Figure 6, adhesion molecules like VCAM1 play a key role in the stable attachment of rolling leukocytes, which is essential for their movement into tissue. A novel contrast agent system model has been created by emulating the behavior of white blood cells when they encounter inflammation (Ferrante et al., 2009). This study aimed to enhance the ability of microbubbles to adhere under the shear stress caused by blood flow, in order to detect inflammation associated with atherosclerosis. In cell culture experiments, microbubbles designed to simultaneously target both P-selectin and VCAM1 showed increased adherence efficiency compared to those targeting only one of these molecules. The hypothesis suggested that, much like the process involving leukocytes, directing the microbubbles towards the P-selectin marker helped capture them on the endothelial cell wall, enabling them to roll along the endothelial cells. Meanwhile, concentrating on VCAM1 enabled the microbubbles to firmly attach to the blood vessel walls, as illustrated in Figure 6 (Deshpande et al., 2010).

Microbubbles have been utilized to evaluate inflammation in different organs, including the heart, colon, and kidneys, by targeting inflammatory markers such as E-selectin, P-selectin, ICAM1, and VCAM1 (Kaufmann et al., 2007a; Bachmann et al., 2006). By employing microbubbles targeting P-selectin in a rat model of myocardial ischemia-reperfusion, researchers were able to detect heart tissue that had undergone recent ischemic events without signs of cell death (Kaufmann et al., 2007b). In a related investigation, sialyl Lewis was associated with microbubbles in a rat model of myocardial ischemia-reperfusion. The substance then bound to the inflamed endothelial cells within the heart tissue, which had recently experienced decreased blood flow (Villanueva et al., 2007). Both studies emphasize the promise of molecular ultrasound imaging as a quick and easy bedside method to detect “ischemic memory” in patients experiencing unusual chest pain. Ischemic Memory refers to the phenomenon where tissues, particularly in the brain or heart, exhibit a form of “memory” after an ischemic event (restricted blood flow that leads to oxygen deprivation) (Aras and Dilsizian, 2007; Villanueva et al., 2007; Taegtmeyer and Dilsizian, 2008). After such an event, tissues may undergo changes in cellular signaling, gene expression, and structural adaptations that make them more susceptible to future ischemic injury or, conversely, potentially more resistant depending on how the tissue adapts (Taegtmeyer and Dilsizian, 2008; Sun, 1999). This memory is thought to arise from cellular and molecular changes that persist after the acute phase of ischemia, possibly influencing the response to subsequent ischemic events or interventions (Aras and Dilsizian, 2007). Moreover, ultrasound imaging aimed at P-selectin could potentially be applicable in diagnosing other inflammatory heart conditions, like myocarditis and transplant rejection. Ultrasound, particularly contrast-enhanced ultrasound molecular imaging, has shown promise in detecting myocarditis, even before functional decline in left ventricular performance is observable. In a study with mice, MB targeted at specific markers, such as CD4^+^ T cells (MBCD4) and endothelial P-selectin (MBPSel), were used for imaging myocardial inflammation and leukocyte infiltration. The study found that molecular imaging with targeted microbubbles provided increased signal detection in moderate and severe myocarditis, correlating with CD4^+^ T-cell infiltration in the myocardium. This technique was sensitive enough to detect endothelial inflammation and leukocyte activity, offering a valuable diagnostic tool for myocarditis, particularly in cases where traditional functional imaging, like left ventricular ejection fraction or strain, did not show significant changes. The ability to image immune cells such as CD4^+^ T cells involved in autoimmune responses may enhance early diagnosis and monitoring of myocarditis (Steinl et al., 2016). A key potential use of molecular ultrasound in medicine is to evaluate the level of inflammation in patients suffering from inflammatory bowel disease. In a pioneering study, researchers were able to observe inflammation at the molecular level in the terminal ileum of genetically modified mice using non-invasive methods. This was achieved by using microbubbles that targeted the mucosal address in cell adhesion molecule 1 (MAdCAM1) (Bachmann et al., 2006).

### 4.4 US for intravascular thrombosis formation

Utilizing molecular ultrasound to visualize blood clot formation shows great potential, not only in diagnosing conditions but also in informing treatment decisions for stroke patients or individuals at higher risk for cerebral embolic disease. Moreover, employing microbubbles in conjunction with ultrasound energy can be an effective method for dissolving blood clots. While research on molecular imaging of blood clots is still in its early stages, various studies have demonstrated that molecular ultrasound can successfully image thrombi in animals by directing contrast agents towards the elements involved in blood clotting. Research conducted by Schumann and colleagues demonstrated that targeted microbubbles adhered to blood clots in the arteries and veins within the cremaster muscle of mice (Schumann et al., 2002). To create these particular agents, the microbubble shell was incorporated with peptides that attach to the Platelet glycoprotein (GP) IIb/IIIa receptor. A related study employed microbubbles coated with peptides aimed at GP IIb/IIIa to identify blood clots in the femoral veins of canines (Wang et al., 2006).

### 4.5 Emerging vascular applications of ultrasound

Beyond thrombosis, ultrasound-triggered drug and gene delivery to the vascular endothelium is now being explored for conditions such as atherosclerosis, vascular inflammation, and restenosis (Sitta and Howard, 2021; Fan et al., 2022). Contrast-enhanced ultrasound is increasingly used in the early detection of vascular pathologies, including endoleaks after endovascular aneurysm repair, assessment of neovascularization in atherosclerotic plaques, and monitoring of angiogenesis in tissue-engineered grafts (Jawad et al., 2016; Bredahl et al., 2017). Furthermore, ongoing clinical trials are evaluating the safety and efficacy of ultrasound-assisted vascular interventions, aiming to enhance precision and minimize reliance on systemic therapies (Dieter and Nanjundappa, 2025; Avgerinos et al., 2021). These developments are moving ultrasound technology from diagnostic imaging toward a platform for minimally invasive, image-guided vascular therapy. Recent technological advances in foam sclerotherapy highlight the importance of bubble size distribution and foam stability for effective and safe vascular interventions. For example, ultrasound-generated foams have been shown to produce smaller, more uniform bubbles and enhanced stability compared to traditional manual methods, as demonstrated by Critello et al. (Critello et al., 2020). These innovations are particularly relevant for the treatment of small veins, where precise occlusion and minimal adverse events are desired. Such advances in foam engineering are expected to make sclerotherapy even safer and more versatile, broadening its clinical applications in vascular medicine (Critello et al., 2020; Critello et al., 2019).

### 4.6 Recent developments in ultrasound-mediated therapies

The field of therapeutic ultrasound has witnessed rapid advancements, with several new modalities reaching clinical or near-clinical translation. High-intensity focused ultrasound (HIFU) and low-intensity pulsed ultrasound (LIPUS) are now being investigated for vascular tissue regeneration, modulation of vascular permeability, and targeted ablation of vascular malformations (Harrison and Alt, 2021; Uddin et al., 2021). In oncology, ultrasound-mediated delivery of chemotherapeutic agents or immunomodulators to tumor vasculature is being refined through the use of smart microbubbles and nanodroplets, enabling site-specific release and reducing systemic toxicity (Fan et al., 2022; Ayana et al., 2022).

## 5 Ultrasound in medicine: current limitations and future direction

Ultrasound technology has evolved from its early applications in diagnostic imaging to encompass a wide array of therapeutic and molecular uses. Despite its numerous benefits, such as non-invasiveness, low cost, and portability, the field of ultrasound, especially in therapeutic and molecular applications, faces several limitations that must be addressed for further advancement.

### 5.1 Current limitations and strategic solutions

One of the most significant limitations of ultrasound is its restricted depth penetration, especially for therapeutic applications (Jensen et al., 2006; Gorny et al., 2005). High-frequency ultrasound provides excellent resolution but is limited to superficial tissues (Gorny et al., 2005). This presents challenges when treating deep-seated tumors or internal organs without damaging overlying healthy tissues (Sun M. et al., 2022; Wu et al., 2023). Advances such as focused ultrasound, phased-array transducers, and adaptive beamforming are improving energy delivery to deeper tissues while minimizing collateral damage (Deng et al., 2021; Goudarzi et al., 2024). Ongoing research into novel coupling materials and alternative acoustic windows may further enhance penetration in anatomically challenging regions.

The biological effects of therapeutic ultrasound can be inconsistent and are highly dependent on parameters such as frequency, intensity, and exposure duration (Chen S. et al., 2022; Quarato et al., 2023). The same ultrasound settings may have different effects on different tissues, posing risks of under- or over-treatment (Chen L. et al., 2022). Standardization through large-scale clinical trials and development of real-time monitoring systems (such as MRI guidance or thermal mapping) can help ensure consistent dosing and outcomes. Personalized treatment algorithms that optimize parameters for individual patient characteristics are also under development.

Microbubbles have revolutionized drug delivery and molecular imaging (Chen L. et al., 2022; Liu et al., 2024), but their short lifespan and need for high-intensity ultrasound for cavitation remain limitations (Critello et al., 2019). Achieving precise targeting and avoiding non-specific adhesion to healthy tissue can also be problematic (Chen L. et al., 2022; Liu et al., 2024; Sanwal et al., 2021). Engineering of longer-circulating, ligand-targeted, or stimuli-responsive microbubbles is ongoing (Lohse and Zhang, 2015; Zylberberg and Matosevic, 2016; Mishra et al., 2025). Techniques such as the use of nanodroplets, low-intensity ultrasound activation, and improved surface modifications are being developed to enhance stability, targeting precision, and therapeutic efficacy.

There is a lack of widely accepted protocols for the clinical use of therapeutic and molecular ultrasound. Variation in equipment, manufacturer parameters, and operator technique leads to inconsistent outcomes and hinders broader adoption. International expert panels and professional societies are working to create consensus guidelines for ultrasound parameters, dosimetry, and clinical reporting. Standardization will facilitate reproducibility, cross-study comparison, and regulatory approval.

While generally safe, therapeutic applications—especially high-intensity focused ultrasound (HIFU)—carry risks such as tissue damage, burns, nerve injury, and hemorrhage (Bachu et al., 2021; Cranston et al., 2021; Lu et al., 2024). Prolonged or excessive exposure can result in unintended thermal and mechanical effects (Filippou et al., 2021).

Safety monitoring technologies—including real-time temperature mapping and acoustic emission feedback—are being integrated into modern devices. Preclinical safety testing and the establishment of safety threshold values are becoming standard in clinical trials.

Regulatory approval processes for ultrasound devices—particularly for new molecular or therapeutic applications—are lengthy and complex, requiring comprehensive demonstration of safety and efficacy (Deshpande et al., 2010). Early engagement with regulatory agencies (such as the FDA and EMA), inclusion of standardized clinical endpoints, and multidisciplinary collaboration in trial design are crucial for streamlining the approval process and expediting clinical translation.

There is no universally accepted framework for measuring key ultrasound parameters (e.g., intensity, frequency, exposure time) (Shaw et al., 2015; Shaw and Hodnett, 2008). Variability across machines and clinics can lead to inconsistent results and limit comparability (Shaw et al., 2015; Chung et al., 1999). The development and validation of international dosimetry standards, reference phantoms, and calibration protocols are underway. Open-source data sharing and harmonized reporting standards will further support reproducibility and safe clinical practice.

Unintended bioeffects, particularly in oncological HIFU applications, raise ethical concerns—including risks of thermal damage, cavitation, and microvascular rupture (Schmid et al., 2020; Fleury et al., 2005). The long-term effects of microbubbles and targeted agents in molecular imaging are not fully understood (Newson, 2008; Uyyala, 2023). Robust ethical guidelines, transparent patient consent procedures, and long-term post-treatment surveillance are necessary. Continued research on biological effects and clear communication of risks will be essential, especially for use in vulnerable populations.

### 5.2 Future directions

1. **Enhanced targeting and specificity**: A key area for future development in ultrasound technology is improving the targeting capabilities of microbubbles and other contrast agents. Advances in molecular biology, particularly in the field of biomarker discovery, will enable the development of more specific microbubbles that can bind to disease-specific molecular markers (Rincon-Torroella et al., 2022; Meng et al., 2021; Langeveld et al., 2021). This will improve the targeting of ultrasound therapies and imaging, enhancing their effectiveness while minimizing side effects. Moreover, the integration of ultrasound with other imaging modalities, such as MRI or CT, could provide complementary information to improve the targeting and accuracy of treatments (Kloth et al., 2021; Moreel et al., 2023).
2. **Personalized treatment protocols**: As more is understood about the cellular and molecular effects of ultrasound, there is an opportunity to develop personalized treatment protocols. Machine learning and artificial intelligence could play a crucial role in analyzing ultrasound data and optimizing treatment parameters for individual patients based on their unique tissue characteristics and disease states (Kloth et al., 2021; Quazi, 2022; Saba et al., 2024; Ahn et al., 2023). This could lead to more effective and tailored treatments, particularly in areas like cancer therapy, where precision is critical.
3. **Integration with other therapies**: The combination of ultrasound with other therapeutic modalities, such as chemotherapy, gene therapy, or immunotherapy, represents an exciting avenue for future research (Lopez et al., 2021; Newman and Bettinger, 2007; Unga and Hashida, 2014). For example, ultrasound-triggered sonoporation could enhance the delivery of chemotherapeutic drugs or genetic material into targeted cells, potentially overcoming issues such as drug resistance (Newman et al., 2001; Yu et al., 2006). Additionally, ultrasound’s ability to promote tissue regeneration could complement other regenerative medicine techniques, such as stem cell therapy (Amini et al., 2020).
4. **Improved bioeffect modeling**: To minimize the risk of adverse effects and optimize treatment outcomes, there is a growing need for better bioeffect modeling (Amini et al., 2020; Dell'Italia et al., 2022; Muratore et al., 2009). Understanding the complex interactions between ultrasound waves and different tissues at the molecular level will allow researchers to predict and control the bioeffects more effectively. Advances in computational models and simulations will be key to developing these predictive tools, making it possible to fine-tune ultrasound treatments for specific applications (Dell'Italia et al., 2022).
5. **Regulatory approval and clinical trials**: As ultrasound technology advances, it will be crucial to conduct rigorous clinical trials to establish the safety and efficacy of new ultrasound-based therapies. Furthermore, achieving regulatory approval for these novel applications will require the development of clear, standardized guidelines for their use in clinical practice. Only with the support of solid evidence and regulatory backing can ultrasound technology fully realize its potential as a mainstream therapeutic modality.

## 6 Conclusion

Ultrasound technology has evolved into a multifaceted tool in modern medicine, not only serving as a diagnostic tool but also holding substantial promise for therapeutic applications. Techniques like sonoporation and microbubble-based therapies have shown potential in enhancing drug delivery, facilitating cellular repair, and improving cancer treatments. In parallel, molecular ultrasound imaging offers significant advances in non-invasive monitoring, allowing for real-time observation of disease processes such as angiogenesis and inflammation. The integration of these two domains—therapeutic ultrasound and molecular imaging—presents a unique synergy. Molecular imaging can guide therapeutic interventions by targeting specific biomarkers, while therapeutic ultrasound enhances the precision and effectiveness of these treatments. As research continues to uncover novel applications and refine these approaches, ultrasound is poised to become an essential tool in personalized medicine, providing a dynamic means to not only monitor but also directly impact disease progression, making it an invaluable asset in future clinical practice.

## Abbreviations

AI, Artificial Intelligence; AFM, Atomic Force Microscopy; ALARA, As Low As Reasonably Achievable; BBS, Bubble-Based Sonoporation; CD4^+^ T cells (MBCD4), Cluster of Differentiation 4+ T cells; CT, Computed Tomography; FDA, Food and Drug Administration; EMA, European Medicines Agency; ICAM1, Intercellular Adhesion Molecule 1; IRS, Irreversible Sonoporation; LOC, Loss of Correlation; MBs, Microbubbles; MAdCAM1, Mucosal Address in Cell Adhesion Molecule 1; MAPKs, Mitogen-Activated Protein Kinases; MEGAhertz (MHz), Measurement of Frequency in Megahertz; MI, Mechanical Index; NBBS, Non-Bubble-Based Sonoporation; PI, Pulse Inversion; ROS, Reactive Oxygen Species; Rho family GTPases, A family of small signaling GTPases; SEM, Scanning Electron Microscopy; TEM, Transmission Electron Microscopy; TMC, Transmembrane Current; VEGFR2, Vascular Endothelial Growth Factor Receptor Type 2; VEGF, Vascular Endothelial Growth Factor; VCAM1, Vascular Cell Adhesion Molecule 1; US, Ultrasound; HIFU, High-Intensity Focused Ultrasound; MPa, Megapascals (Unit of Pressure)
